# Advance Care Planning among People Living with Dialysis

**DOI:** 10.3390/healthcare4010017

**Published:** 2016-03-03

**Authors:** Barbara A. Elliott, Charles E. Gessert

**Affiliations:** 1 Department of Family Medicine and Community Health, University of Minnesota Medical School, 1035 University Drive, Duluth, MN 55812, USA; 2 Essentia Institute of Rural Health, 407 East Third Street, Duluth, MN 55805, USA; cgessert@yahoo.com

**Keywords:** advance care planning, advance directives, end-stage-renal-disease, dialysis, end-of-life care

## Abstract

*Purpose:* Recent nephrology literature focuses on the need for discussions regarding advance care planning (ACP) for people living with dialysis (PWD). PWD and their family members’ attitudes toward ACP and other aspects of late-life decision making were assessed in this qualitative study. *Methodology:* Thirty-one interviews were completed with 20 PWD over the age of 70 (mean dialysis 34 months) and 11 family members, related to life experiences, making medical decisions, and planning for the future. Interviews were recorded, transcribed and analyzed. *Findings:* Four themes regarding ACP emerged from this secondary analysis of the interviews: how completing ACP, advance directives (AD), and identifying an agent fit into PWD experiences; PWD understanding of their prognosis; what gives PWD lives meaning and worth; and PWD care preferences when their defined meaning and worth are not part of their experience. These PWD and family members revealed that ACP is ongoing and common among them. They did not seem to think their medical providers needed to be part of these discussions, since family members were well informed. *Practical implications:* These results suggest that if health care providers and institutions need AD forms completed, it will important to work with both PWD and their family members to assure personal wishes are documented and honored.

## 1. Introduction

End of life care, including hospice and palliative care, have become important topics of discussion in society, in medical care, and specifically in the care of people living with dialysis (PWD) [1]. Over the past 20 years, efforts have emphasized the completion of advance directives (AD) to guide the use of medical interventions that are perceived to be life-extending [2]. More recently, discussions have focused on the delivery of patient-centered care and advance care planning (ACP) [3,4]. The discussion of end-of-life care is expanding to include patient and family wishes for this phase of life [5,6].

Consistent with these broad advances in end-of-life care, nephrology is changing as well. The recent nephrology literature includes professional society recommendations [7], clinical practice guidelines [8], advance care planning discussions [9], and research focused on shared decision making [10]. Today, clinicians are called upon to talk with PWD and families about their goals and preferences for care late in life and how medical interventions can best support PWD in fulfilling their goals in both living and dying.

In the past two decades, qualitative work has reported discussions of advance care planning and end of life care experiences with PWD [11,12,13,14]. Over time, the results of these studies have provided the basis for further research on PWD decision making near the end of life. However, the findings of these studies have not been fully integrated into clinical practice. As early as the early 1990s, patients expressed the desire for early and honest discussions with their nephrologists about their prognosis [15]. However, as recently as 2010, fewer than 10% of patients reported having these discussions with their nephrologist in the previous 12 months [3].

Most recent literature in this area addresses whether, how, and who should have the conversations with PWD and their families regarding these topics, as well as what the discussions should actually include. Chronologically, Kirchhoff and colleagues [16] reported the findings of a clinical trial that tested a facilitated advance care planning intervention. The purpose of the study was to learn if surrogates’ understanding of patient goals for future care was improved when involved with a disease-specific planning process. Kirchhoff and her colleagues based this research on the La Crosse experience with Respecting Choices’ Advance Care Planning [17]. The intervention approached the advance care planning discussions with nephrology patients and family members using five steps: assessing the patient’s understanding of their current medical condition and perception of living well; exploring the experiences that have impacted goals for future medical care; discussing specific treatment choices the patient would be likely to experience; preparing a disease specific document noting acceptable or unacceptable burdens and outcomes; and reviewing the discussion for potential future discussions. It is important to note that Kirchhoff and colleagues’ intervention discussions [16] were led by trained facilitators, who were nurses, social workers or chaplains.

In 2012, Davison published suggestions for Advance Care Planning (ACP) in patients with chronic kidney disease [18]. She identified important elements for an ACP conversation with PWD, based on her review of the literature, making recommendations similar to those in the Kirchhoff study, but her suggestions did not include the preparation of a disease specific document to guide future health care decisions.

Most recently, Atul Gwande’s best selling book, Being Mortal [19], has brought these discussions to a societal level. Although it is not focused specifically on care of PWD, Gwande’s guidelines parallel and extend those within the specialty writings. His suggestions are that families need to discuss these questions with their loved ones in an ongoing way to learn what a person prefers as their disease progresses. He suggests discussions that ask these questions: what do you expect will happen to you over time with this disease; what is important to you now (to confirm personal values and goals); what is the bottom line—what changes and losses are you willing to tolerate (e.g., “As long as I can eat ice cream and watch football on TV, keep me going”); and who should speak for you, if and when you cannot speak for yourself (emphasizing the need to include these people in the discussions, too). Similar to Davison’s work [18], Gwande’s recommendations emphasize that these conversations among family members need not include the preparation of the document or form.

These three authors have suggested parallel models to guide discussions with patients and family members or surrogates: they each describe complementary and overlapping processes to learn PWD goals and preferences for health care late in life. Employing these authors’ work, ACP can be broadly defined to include all discussions and plans PWD and families have for end of life care. Kirchhoff’s clinical trial documented that the role of the family surrogates was enhanced with facilitated discussions [16]. Research in this area is ongoing, including a current study by Eneanya *et al*, investigating shared decision making in end stage renal disease. The results of that trial will further clarify how best “to establish preferences and goals at end of life for” PWD [10] (p. 30).

Despite these advances, questions remain about whether PWD and family members (or agents) want to or can talk about these topics with their nephrologists and health care professionals, and their thoughts regarding documenting these ideas. A few qualitative studies have explored these issues, with Goff *et al.* [9] reporting that PWD want better communication with their nephrologists—noting that they have felt excluded from discussions about their health care. In addition, Goff and colleagues reported that the patients wanted both their nephrologists and social workers involved in these conversations, and that the patient’s life experiences, personalities and relationships affected their perspectives regarding ACP.

Overall, the findings to date imply that “complex end-of-life care planning interventions may be more effective in meeting patients’ preferences than written documents alone” [20] (p. 1000). However, the extent to which PWD and their families regard continuing dialysis and other interventions as medical decisions has not yet been investigated. PWD may not think of late-life decisions as medical in nature: many PWD are primarily concerned with living a good life as long as possible [21,22]. Unanswered areas for study include an improved understanding of how dialysis fits into PWDs’ living that good life, what makes life worth living through PWDs’ eyes, and how medical care (including dialysis) is part of living and dying.

The findings of the current study present insights regarding these remaining questions, based on interviews with a group of PWD and their family members. Between 2007 and 2009, a series of interviews of PWD and their family members were completed to learn how they made difficult decisions about initiating, continuing, or stopping dialysis. Qualitative research methods were utilized to understand the factors that patients and families regarded as important in their decision-making. The principal findings from the study were concentrated in three broad arenas of the participants’ experiences with dialysis: adjustment to life on dialysis; reflections on the end of life; and finding meaning in a life on dialysis [23]. Additional insights regarding PWD changing quality of life on dialysis and about their spiritual needs were reported separately [22,24].

For the current study, a secondary analysis of these interview transcripts was completed to examine the attitudes of PWD and their families toward ACP and other aspects of late-life decision making.

## 2. Materials and Methods

This study was conducted in accordance with the Declaration of Helsinki, and the protocol was reviewed and approved by both the Essentia Health IRB (Study Number: #06-05-05), and the University of Minnesota IRB (Study Number: 0507S71914). All participants (PWD and a family member) were invited and then gave their written informed consent for inclusion before they participated in the study. The informed consent allowed for analysis of factors important to patients and families in deciding to start, continue or stop dialysis.

### 2.1. Accrual and Data Collection Methods

People with end stage renal disease (ESRD) over the age of 70, who had been on dialysis for at least 6 months, and their close family members were recruited to participate in this study. Potential participants who met these inclusion criteria were identified by the St. Mary’s Duluth Clinic (SMDC) Nephrology Department in Duluth, Minnesota. The list of potential participants was screened by the SMDC nephrologists to select individuals who, in the view of their physicians, would be able to participate meaningfully in the study (*i.e.*, they were living without cognitive impairment, debility, or other relevant limitations). The Nephrology Department then mailed an information package about the study to the potential participants. A brief introduction to the study was included with an invitation to indicate interest by calling study staff or returning an enclosed form.

Study staff contacted potential participants who expressed interest in the study. They confirmed that the PWD and/or family members met the inclusion criteria, and then provided additional information and answered questions about the study. For those who agreed to participate, appointments were made for the research team to meet with the PWD and/or family members, either in the home or where the PWD received care.

As each interview began, the purpose of the study was explained; written informed consent was obtained; and a brief questionnaire on demographic and contact information was completed. PWD and their family members were interviewed separately when both participated. Family members were interviewed without PWD voice in nine cases, after the PWD had discontinued dialysis and was no longer able to respond. The interviews used an open-ended, qualitative format and were audio-recorded. An Interview Guide kept the discussion focused on seven themes: (a) PWDs’ and family members’ experiences with kidney disease and dialysis; (b) how they have managed up to now; (c) what has affected the course of their disease; (d) reasons to continue/forego dialysis; (e) any advance care planning to date; (f) other topics participants would like to discuss; and (g) participants’ advice for improving care of people receiving dialysis. In addition, pre-determined probes (follow-up questions) were used.

### 2.2. Data Analysis

Consistent with qualitative research methods, the data collection and analysis for the original work was simultaneous and continuous [25]. Draft transcripts were proofed against the audio recordings of the interviews [26]. Data analysis was started as soon as the transcripts from the first 3–4 interviews were available. Participant accrual and data collection were continued until “saturation” had been achieved in the data analysis, at which time accrual was stopped [26]. While a total of up to 40 cases was anticipated, the investigators felt that saturation had been achieved after 31 interviews.

For this secondary analysis, these proofed transcripts were restudied. The analytic process was essentially inductive in nature, with codes and categories emerging from the language and ideas of the participants. The data were indexed, coded and interpreted using the steps outlined by Frankland and Bloor [27]. The transcripts were read by the investigators for a “sense of the whole” independently, and they identified codes to attach to words, phrases, and blocks of text. These investigators then met, developed a unified system of coding by integrating and reconciling their coding, terms, and identifying emerging categories (groups) of codes. These steps were important in view of the difficulty that different readers of the same transcripts can have in arriving at the same conclusions [26,28]. Q.S.R. NUD*IST V5, a qualitative software program, assisted in the comparison process. These transcripts and codes generated themes regarding the experiences of these PWD and their family members related to questions of ACP while living with dialysis.

Thirty-one interviews with PWD and their family members were completed. To better understand decision making at the end of life, one-third of the sample included PWD who were actively discontinuing dialysis. In nine of these eleven cases, only the family member could be interviewed, as the PWD was no longer able to respond. Thus, the thirty-one interviews included twenty PWD and eleven family members (seven children and four spouses). As Table 1 indicates, PWDs’ ages ranged between 70 and 100 years, with a mean of 80.6 years. The patients were all Caucasian; and their average Charlson score was 6.42. Fifteen PWD were male, and twelve were female.

## 3. Results

Four themes emerged in the analysis of the interviews with PWD and their family members regarding PWD thoughts about ACP. These themes included four dimensions of ACP as identified by the recent literature that has specified what ACP conversations need to include: how completing advance care planning, advance directives, and identifying an agent fit into PWD experiences; PWD understanding and experience of their prognosis; what gives PWD lives meaning and worth; PWD care preferences when their defined meaning and worth are no longer part of their lived experience.

### 3.1. Where ACP, ADs and the Role of an Agent Fits into PWDs’ Experiences

Few PWD reported clear knowledge of having advance directives or earlier discussions with health care providers about what interventions they would choose if they were to need aggressive medical care.

- I: Do you recall if you put any instructions into your living will at all? P: Not that I know of. The only thing—my oldest son, he’d be the [decision maker]. (PWD 22) [29]
- I: Do you have an advance directive, like a living will, documenting your wishes? P: No, not right now. We’ve talked about it, but we don’t have anything written down. (PWD 15P)
- Just a verbal understanding. (PWD 3)
- We talked about that for a long time. We had that all planned ahead. We just hadn’t put it down in writing. (PWD 15)

There were exceptions to the majority’s approach to completing the forms. There was one PWD that responded this way:

- I: So in terms of getting sicker, you haven’t planned ahead. It sounds like because you don’t like to think about that P: I just feel that the doctors will take care of it, and I don’t worry about it”. (PWD 17)

Another was at the other extreme:

- I can’t imagine anyone not having an advance directive. I mean it just blows my mind. I mean it’s like HELLO, who do you think is gonna make these decisions? (PWD 5)

Nonetheless, PWD were very clear about what they did want and that they had discussed their wishes with family members. They also reported that these conversations were ongoing and routine.

- I: Are your daughters familiar with your thoughts that you don’t want to be a vegetable and that kind of thing? P: Oh, yes, definitely! We have talked about this. They agree with me. (PWD 21)
- We didn’t have any real recent conversations. It was just that we’d always known what she wanted because we had had several talks over the years. (PWD 23)
- I also had a lot of talks with mom, and I know what she wanted, even then it was very hard, to make that decision to take the tube out. It was just doing the right thing. (PWD 23)
- That [was] all planned ahead not to prolong his suffering. (PWD 4)
- She would never want to be on a feeding tube as they suggested we do. Absolutely she had signed off and we had discussed that many, many times. (PWD 2)

### 3.2. How PWD Understand Their Prognosis

PWD’s reported living “as normal lives as possible” through the early and stable times of dialysis: “Ya, know, it’s like I say going to work. I’m reading and [dialysis] is my half-time job”. (PWD 19) However, PWD also clearly knew they were living in special circumstances:

- I didn’t wish to be on dialysis, but it’s better I would think than dropping dead; yeah you can go to dialysis and live, or you can sulk about it and drop dead. (PWD 14)
- Dialysis is a form of life support. (PWD 18)

As they lived with dialysis longer, the burden of the disease and the dialysis process were reflected in the comments. They recognized the changes in their health and the acceleration of symptoms and health events:

- I would say the last six months or so, things just [have] gotten tougher and tougher. (PWD 6)
- The last month, maybe six weeks or so, it just got really bad really fast. It was like one thing after another just kept happening and happening and happening. (PWD 3)
- He just felt that to be going through the dialysis [and] having the pacemaker put in, that he would’ve expected to feel better. If he couldn’t feel any better than that he’s ready to go. (PWD 5)

Family members also observed that PWD’s know the eventual outcomes of living with dialysis: “Of course she knew it would eventually get too much for her”. (PWD 24)

### 3.3. What Gives PWDs’ Lives Meaning and Worth

While living with dialysis, PWD find value in life and meaning in their existence. Their descriptions of these dimensions of their lives were both philosophical and practical:

- I know we’re only on this earth for a specified period of time and whether that is 10 years or 100 years of age, as I grow older, I know I’m getting closer to that magic day whatever it might be. (PWD 11)
- I have seen some of these people come into the hospital. I mean I THINK I AM LUCKY, I feel anyway, I needed to express that. (PWD 21)
- I am just so appreciative of my being the way I am compared to [the others at dialysis]. Just one day at a time. (PWD 15)

One important way of defining whether life was worth living was based on their sense of their quality of life.

- P: My quality of life is important. I: What kinds of things do you associate with a good quality of life? P: Just being alive and being able to recognize what’s going on. (PWD 12)
- I don’t want to live if I can’t take care of myself a little bit. (PWD 24)
- For her, quality of life [is] if she can enjoy food, enjoy her family, be around those that she loves, and have some intelligent conversation. That’s quality of life. (PWD 2)

Another important way of defining worth and meaning was through an acknowledged faith and relationship with the sacred.

- Well, if you have [faith], there’s nothing to fear. No fear of death. I’m ready to go anytime. (PWD 8)
- I: Let me ask about how your faith has helped you with it all? P: Yeah, I’m not scared at all. But I still want to live. (PWD 27)

### 3.4. What PWD Want When They Know Their Desired Life is no Longer Possible

Many of the PWD acknowledged that they knew a time would come when they could no longer live with the quality of life and wellbeing they desired, and they talked about this easily. They reflected that they knew their lives would end, and that they had options regarding how they wanted the end of their lives to be handled.

- It’s possible [I will get as sick as others at dialysis]. If that’s what happens, I’ll do what I have to do: then I die. (PWD 12)
- No heroic measures. No resuscitation. No breathing machines. (PWD 5)
- If I get real helpless and can’t do anything, then I don’t want to be just kept alive on machines. (PWD 9)
- P: Get me to the hospital, but, if there’s no hope and if I’m gonna be a vegetable, let me go. I: Many people use that expression ‘vegetable’ what do you take it to mean? P: When a person is no longer able to function, and it’s not fair to your family. That’s the way I look at it. (PWD 21)
- No, I don’t want anything like that. No, it’s just another prolongation of your death. There are some things that I don’t want to be put on, but kidney dialysis is okay. (PWD 8)
- She was always very clear that she didn’t ever want a breathing tube and she didn’t want all this extra stuff, and if it was her time, it was her time. (PWD 23)
- When I become a burden on the rest of the family, or if it interrupts their family life, then THAT is the point that I WANT to be removed so that I’m not a burden anymore but can go on through hospice. We’ve discussed this. I have no qualms about it. (PWD 11)

## 4. Discussion

The results of these interviews with PWD and their family members revealed that these PWD had done extensive advance care planning and had careful conversations with their close family member—but they were not necessarily sharing these wishes with their health care providers or documenting their wishes. Most PWD were aware that they had been asked about completing an AD by their nephrologist, but they were not motivated to do so. They had no energy for completing these forms, despite being very clear about their prognosis, their desired quality of life, and how they wanted to be cared for when that quality of life was no longer available.

These PWD reported that they had carefully and consistently discussed their wishes with their family members. PWD trusted that their family would be with them to the end; they knew their family would be present to interpret circumstances and make the medical care decisions. In addition, these familial relationships were described as a key aspect of the PWD lives: they were so connected with their families that PWD said that when their care became a burden for their family members, they would discontinue dialysis.

Additional insights gained from these interviews reveal that the conversations these families were having paralleled those recommended in the literature: they specifically reported answers to the questions raised by Kirchhoff [16], Davison [18] and Gwande [19]. First, PWD were informed about their prognosis and how a life supported by dialysis changes over time. They knew this through their experiential learning at the dialysis centers and their own bodies’ experiences. This reality was not learned from conversations with health professionals.

In addition, the values undergirding these PWD lives and their preferences for end of life care were well understood. Participants reported the common desire to be with their family members as long as they were able to function with a level of independence. They did not want to live any longer than their personal definition for quality of life and faith allowed, and they reported that they had clearly spoken with their family and loved ones about this desire. Most PWD did talk openly and freely about what they wanted at the end of life. Dialysis was fine, but (interestingly) other machines were not to be used to keep these PWD alive into the future. Some remained completely focused on today, as if broader discussions would bring the problems “too close for comfort”.

As has become evident from the literature [16,18,19], the essential aspect of ACP is the conversation about what makes life worth living and what the person would want when the things that make life worth living cannot be attained. This conversation reveals personal, existential, and spiritual values. Filling in the AD form does not flow automatically from these conversations, although these insights do inform the person’s end-of-life desires. These PWD and family members definitely reported having the conversations, but were not generally completing the forms.

These results have clinical implications. It is clear that these PWD separated living their lives from their experience of medical care. Respecting PWD and family members’ decisions to determine their life course is part of caring for our patients. Nonetheless, knowing PWD desired medical interventions is also important; gaining that information will strengthen the role of medical care in end-of-life decision making.

These findings suggest a way to approach the completion of AD, based on the revelation that ACP is ongoing in family settings. If it is explained that completion of the AD forms actually benefits the family members when decisions need to be made, they may be persuaded to complete the forms. From our experience with these interviews, and Kirchhoff’s clinical trial [16], these conversations do not need to be done or completed by the nephrologist; a skilled facilitator can create the relationship that allows the conversation and results in completed forms.

The results reported here do need to be read with caution. This qualitative study interviewed a small number of PWD and their family members who were over 70 years of age and lived with fragile health, although there were able and interested to have these conversations. They all also resided in the Midwest (USA) and were Caucasian, of the majority culture. Their circumstances reflect a large portion of our increasing dialysis population, but only a part of the group. In addition, the findings presented here are the result of a secondary analysis of these interview transcripts; it is possible that additional interviews would have expanded the insights specific to ACP and AD completion. Nonetheless, these findings offer suggestions for working with PWD in similar circumstances around ACP issues.

## 5. Conclusions

The comments these PWD and family members shared with us revealed that how people prefer to live their lives is a personal, existential, and family experience. They have ongoing and common discussions about end-of-life issues and preferences, and the topics they reportedly discuss parallel those identified in the literature for ACP discussions. Many participants did not seem to think their medical providers needed to be part of the discussions regarding their life preferences and ACP intentions since their family members were well informed. These results imply that if health care providers and institutions need AD forms completed, it may be important to work with both PWD and their family members to assure personal wishes are documented. Future research and clinical experience are needed to determine the best ways to accomplish this.

## Figures and Tables

**Table 1:** Demographics of people living with dialysis (PWD) in study.

Characteristic	N (%) Answering	N in Response
Race-Caucasian	27 (100%)	27
Gender: Male	15 (56%)	27
Age	80.6 years (Range: 70–100)	27
Marital status		
Married	18 (66%)	27
Widowed	8 (30%)	
Never	1 (4%)	
Quality of Life		
Best possible	2 (11%)	18 of 27
Good	10 (56%)	
Fair	4 (22%)	
Poor	2 (11%)	
Worst possible	0	
Months receiving Dialysis (Mean)	34	26 of 27
Charlson Score	6.42 (Range: 2–12)	24 of 27
Interviews *	PWD	Family Member
PWD only	18	0
PWD and Family	2	2
Family only	0	9
Totals	20	11

* N = 31 individual interviews: 27 PWD were enrolled, 20 PWD were interviewed, eighteen without family member contact; nine of eleven participating family members were interviewed without their incapacitated PWD following decision to forego dialysis.
